# SLC25A28 Ameliorates Hyperoxic Lung Injury by Improving Mitochondrial Oxidative Phosphorylation in Alveolar Epithelial Cells

**DOI:** 10.3390/ijms27083357

**Published:** 2026-04-08

**Authors:** Tao Lu, Shi-Qi Chen, Shu-Hong Li, Sheng-Peng Li, Ya-Xian Wu, Qing-Feng Pang, Dan Chen

**Affiliations:** Department of Physiopathology, Wuxi School of Medicine, Jiangnan University, Wuxi 214122, China

**Keywords:** bronchopulmonary dysplasia, SLC25A28, mitochondrial iron, mitochondrial OXPHOS, alveolar epithelial cell, SS-31

## Abstract

Mitochondrial dysfunction plays a central role in the pathogenesis of bronchopulmonary dysplasia (BPD). Solute carrier family 25 member 28 (SLC25A28) is an iron transporter located in the inner mitochondrial membrane. In this study, we aimed to explore the role and underlying molecular mechanisms of SLC25A28 in BPD. Hyperoxia (85% O_2_) was used to establish a neonatal murine model of BPD, and mouse lung epithelial cells (MLE-12 cells) were used in vitro. SLC25A28 expression and activity were downregulated under hyperoxic conditions, both in vivo and in vitro. SLC25A28 overexpression restored hyperoxia-induced mitochondrial oxidative phosphorylation (OXPHOS) dysfunction, and further enhanced the proportion of Ki67-positive cells by 37% (*p* < 0.05) and increased migration by 33% (*p* < 0.01) in MLE-12 cells. In contrast, SLC25A28 knockdown exacerbated these impairments in MLE-12 cells, with reduced the proportion of Ki67 positive cells by 71% (*p* < 0.01) and a 35% reduction in the migration rate. SLC25A28 was also knocked down in vivo, which further aggravated alveolar simplification in BPD mice. Furthermore, the mitochondrial-targeted peptide SS-31 could potentially interact with SLC25A28 and preserve its protein abundance. SS-31 administration mitigated hyperoxia-induced alveolar simplification, with the radical alveolar count (RAC) increasing by 28% (*p* < 0.05) and the mean linear intercept (MLI) decreasing by 20% (*p* < 0.001). In summary, this study revealed that SLC25A28 ameliorated hyperoxic lung injury by improving mitochondrial OXPHOS in alveolar epithelial cells, suggesting that it may serve as a potential therapeutic target for BPD.

## 1. Introduction

As the most common and clinically significant chronic lung morbidity in premature infants, bronchopulmonary dysplasia (BPD) is primarily distinguished by arrested alveolarization and abnormal pulmonary vascular development [1]. Preterm infants with BPD are at high risk of early mortality, and survivors often suffer from lifelong complications such as retinopathy, poor neurodevelopment, and persistent respiratory disorders, which significantly compromise their quality of life [2]. The prevalence of BPD according to the NIH 2001 and NICHD 2018 definitions is 52.4% and 23.9%, respectively [3]. Clinically, prenatal glucocorticoids and postnatal surfactants are typically administered to promote lung development in preterm infants. However, the incidence of BPD continues to increase [4]. To date, no specific or effective strategies for the prevention or treatment of BPD have been established [5]. Therefore, the identification of novel therapeutic targets and the development of effective treatment strategies are urgently needed.

Iron is an essential trace element for mammalian cells. Mitochondria are central sites of cellular iron metabolism, utilization, and storage [6]. Mitochondrial iron is utilized for the synthesis of heme and iron–sulfur clusters (Fe-S), which are indispensable for fundamental biological processes including DNA synthesis and oxidative phosphorylation (OXPHOS), through which the electron transport chain (ETC) generates a proton gradient to drive adenosine triphosphate (ATP) production [7]. Postnatal pulmonary type II alveolar epithelial cell (AEC2) proliferation and growth are highly dependent on an adequate energy supply [8], and OXPHOS is essential for meeting these high energy demands [9]. Abnormal mitochondrial iron levels have been detected in bleomycin-induced pulmonary fibrosis, and cigarette smoke-induced bronchitis and emphysema [10,11]. Furthermore, high oxygen supplementation causes Fe-S cluster destabilization, and impairs ETC function [12]. These findings indicate that iron homeostasis is critical for maintaining OXPHOS capacity and mitochondrial function in hyperoxia.

Mitochondrial iron relies on specific transporters, with solute carrier family 25 members 37 (SLC25A37) and 28 (SLC25A28) being key players [13,14]. SLC25A37 is predominantly expressed in erythroid progenitors and is mainly involved in hemoglobin synthesis [15]. In contrast, SLC25A28 is ubiquitously expressed in non-erythroid tissues and serves as the primary mitochondrial iron importer required for basal Fe-S cluster assembly. It has been reported that SLC25A28 and SLC25A37 are required for liver regeneration and cell proliferation in mice [16]. Furthermore, microglial SLC25A28 deficiency mitigates spinal cord injury in mice [17]. Loss of SLC25A28 exacerbates the development of metabolic dysfunction-associated steatotic liver disease (MASLD) on a low-iron diet [18]. Given that BPD predominantly involves injury to AECs, which belong to a strictly non-erythroid lineage, and SLC25A28 is reported to be the primary transporter maintaining mitochondrial iron homeostasis in non-erythroid cells under pathological conditions [13]. We hypothesized that SLC25A28, rather than the erythroid-specific SLC25A37, may play a critical role in the pathogenesis of BPD.

As mitochondrial dysfunction plays central role in the pathogenesis of BPD, and hyperoxia impairs OXPHOS in AEC2s (reducing ATP, disrupting mitochondrial membrane potential (MMP), and compromising ETC), pharmacological interventions capable of restoring mitochondrial OXPHOS hold significant therapeutic promise. SS-31 (elamipretide), a novel mitochondrial-targeted antioxidant peptide, has emerged as a potent agent for the treatment of diseases associated with mitochondrial dysfunction, including cardiac and kidney disorders [19,20]. Moreover, SS-31 localizes to the inner mitochondrial membrane to stabilize the cristae structure. Given that SLC25A28 is an inner membrane transporter critical for iron homeostasis, we hypothesized that SS-31 exerts its protective effects by modulating SLC25A28. Previous studies have shown that SS-31 restores MMP, improves ETC efficiency, and increases ATP content [21]. Although the beneficial effects of SS-31 have been demonstrated in respiratory conditions, such as protecting against pulmonary fibrosis [22] and attenuating pulmonary arterial hypertension [23], its potential efficacy in mitigating hyperoxia-induced BPD remains unclear.

In this study, we explored the roles and molecular mechanisms of SLC25A28 in BPD. SLC25A28 overexpression alleviated mitochondrial damage and promoted the proliferation and migration of MLE-12 cells under hyperoxic conditions. In contrast, SLC25A28 knockdown exacerbated hyperoxia-induced MLE-12 cell injury. Additionally, SLC25A28 knockdown aggravated alveolar simplification in BPD mice. Furthermore, SS-31 potentially interacted with SLC25A28 to preserve its abundance. SS-31 administration mitigated hyperoxia-induced alveolar simplification. In summary, this study revealed that SLC25A28 ameliorated hyperoxic lung injury by improving mitochondrial OXPHOS in AECs, highlighting it as a potential target for BPD treatment.

## 2. Results

### 2.1. The Reduction in SLC25A28 Expression and Activity Is Accompanied by Mitochondrial Damage in Lung Tissues of BPD Mice

**Figure 1:**
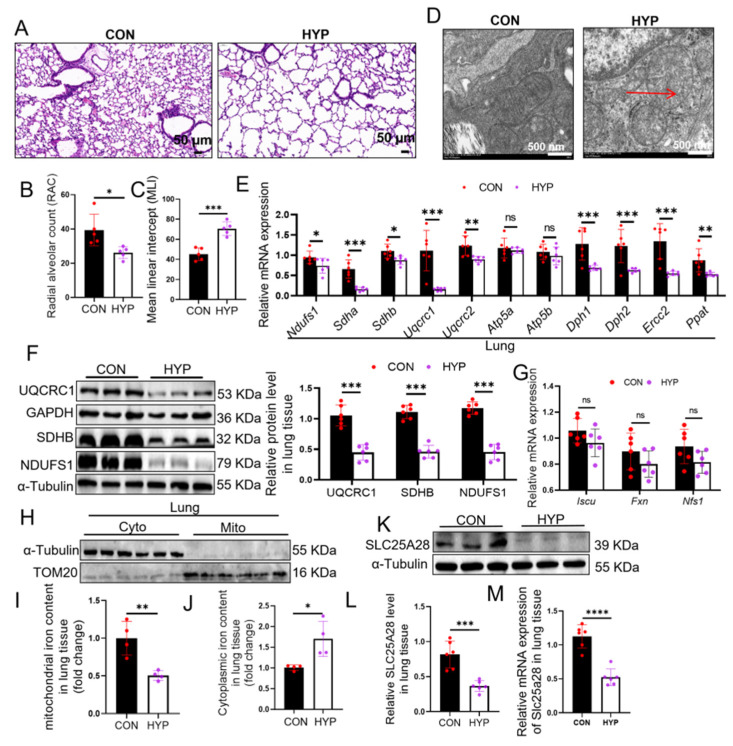
The reduction in SLC25A28 expression and activity is accompanied by mitochondrial damage in lung tissues of BPD mice. (**A**) Representative H&E staining of lung tissues. Scale bar, 50 µm. (**B**) RAC evaluation (*n* = 5 mice per group). (**C**) MLI evaluation (*n* = 5 mice per group). (**D**) Representative TEM images of lung tissue sections, the red arrows indicate the disappearance of mitochondrial cristae. Scale bar, 500 nm. (**E**) Relative mRNA levels of genes involved in mitochondrial OXPHOS (*n* = 6 mice per group). (**F**) Relative protein levels of NDUFS1, SDHB and UQCRC1 (*n* = 6 mice per group). (**G**) Relative mRNA levels of genes related to Fe-S cluster synthesis (*n* = 6 mice per group). (**H**) Western blots showing TOM20 and α-Tubulin expression in the isolated mitochondrial (Mito) and cytosolic (Cyto) fraction from the lung tissue, respectively (*n* = 6 mice per group). TOM20 was used as a marker for Mito fraction and α-Tubulin as a marker for Cyto fraction. (**I**) Mitochondrial iron content in lung tissues (*n* = 4 mice per group). (**J**) Cytosolic iron content in lung tissues (*n* = 4 mice per group). (**K**) Representative images of Western blotting. (**L**) Relative SLC25A28 protein level in lung tissues (*n* = 6 mice per group). (**M**) Relative SLC25A28 mRNA level in lung tissues (*n* = 6 mice per group). Data were expressed as the mean ± SD. * *p* < 0.05, ** *p* < 0.01, *** *p* < 0.001, ns, not significant. BPD, bronchopulmonary dysplasia; NDUFS1, NADH:ubiquinone oxidoreductase core subunit S1; SDHB, succinate dehydrogenase complex, subunit B; UQCRC1, ubiquinol-cytochrome c reductase core protein 1; TOM20, translocase of outer mitochondrial membrane 20; SLC25A28, solute carrier family 25 member 28; TEM, Transmission Electron Microscopy; OXPHOS, oxidative phosphorylation; Mito, mitochondrial; Cyto, cytosolic; CON, control; HYP, hyperoxic. The black columns represent the CON group, and the white columns represent the HYP group. **** *p* < 0.0001.

To mimic hyperoxia-induced BPD, a mouse model was established by exposure to 85% O_2_. Alveolar simplification was found in lung tissues of BPD mice (Figure 1A), with a significant decrease in radical alveolar count (RAC) by 34% (*p* < 0.05) and a significant increase in mean linear intercept (MLI) by 56% (*p* < 0.001) (Figure 1B,C). Moreover, pulmonary mitochondria in BPD mice appeared round and swollen, with disrupted or absent cristae (Figure 1D). In addition, the mRNA levels of the key genes involved in OXPHOS were decreased in lung tissues of BPD mice (Figure 1E). In addition, hyperoxia downregulated the expression of key subunits of ETC (UQCRC1, SDHB and NDUFS1 are subunits of Complex III, Complex II, and Complex I, respectively), which were all belong to Fe-S (Figure 1F). To understand how hyperoxia affects Fe-S cluster synthesis, we examined the key proteins involved in this process. As shown in Figure 1G, there were no statistically significant differences in the mRNA levels of key genes involved in Fe-S cluster synthesis. Iron is an important source for the synthesis of Fe-S cluster proteins. Therefore, we further examined iron levels in lung tissues. As shown in Figure 1H–J, hyperoxia reduced mitochondrial iron levels while increasing cytosolic iron levels. SLC25A28 and SLC25A37 mediate iron transport into the mitochondria. We found SLC25A28 as the gene with the most markedly downregulated in lung tissues of BPD mice when compared with SLC25A37 (Figure S1A). We then focused on SLC25A28, and this result was then further verified by Western blot and immunofluorescence and confirmed the reduced expression of SLC25A28 in BPD lung tissues (Figure 1K–M and Figure S1B,C). These results highlighted the possible effect of SLC25A28 on hyperoxia-induced mitochondrial damage in BPD mice.

### 2.2. Hyperoxia Reduces the Expression and Transport Activity of SLC25A28, Concurrent with Mitochondrial Dysfunction in Mouse Alveolar Epithelial-12 (MLE-12) Cells

**Figure 2:**
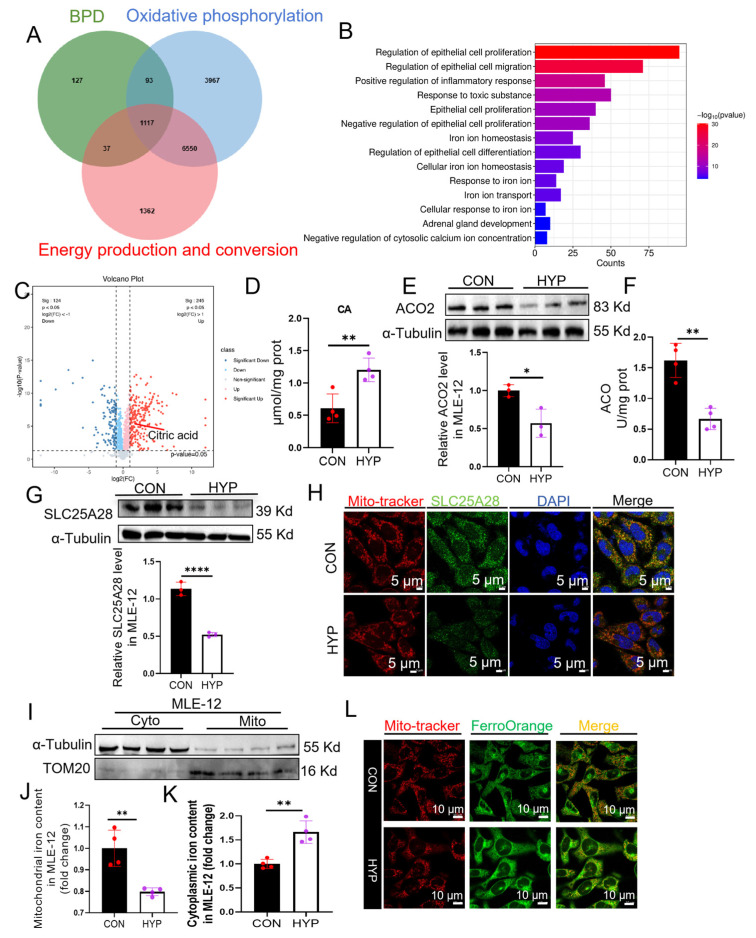
Hyperoxia leads to reduced expression and transport activity of SLC25A28, concurrent with mitochondrial dysfunction in MLE-12 cells. (**A**) Venn diagram including BPD, Oxidative phosphorylation and Energy production and conversion datasets. (**B**) KEGG enrichment analysis of the intersection genes. (**C**) Volcano diagram of differential mitochondrial metabolites. The dashed lines represent the threshold values for statistical significance (e.g., *p* < 0.05 and |log2FC| > 1). The red dots represent significantly upregulated metabolites (HYP vs. CON), the blue dots represent significantly downregulated metabolites (HYP vs. CON), and the grey dots represent non-significant genes. These different color dots are simply part of a standard scatter bar plot. The scatter dots within the bars represent individual biological replicates for each corresponding group. (**D**) Content of citric acid in MLE-12 cells (*n* = 4 independent experiments). (**E**) Relative ACO2 protein level (*n* = 3 independent experiments). (**F**) ACO activity in MLE-12 cells (*n* = 4 independent experiments). (**G**) Relative SLC25A28 protein expression (*n* = 3 independent experiments). (**H**) Immunofluorescence co-staining of SLC25A28 and Mito-tracker in MLE-12 cells. SLC25A28 (green), Mito-tracker (red) and DAPI (blue) (scale bar, 5 μm; *n* = 3 independent experiments). In the merged images, the yellow and orange color indicates the co-localization of Mito-tracker (red) and SLC25A28 (green). (**I**) Representative images of TOM20 and α-Tubulin expression in the isolated mitochondrial (Mito) and cytosolic (Cyto) fraction from the MLE-12 cells, respectively (*n* = 4 independent experiments). TOM20 was used as a marker for Mito fraction and α-Tubulin as a marker for Cyto fraction. (**J**,**K**) Iron contents in the Cyto and Mito fractions isolated from the MLE-12 cells (*n* = 4 independent experiments). (**L**) FerroOrange and mito-tracker staining. FerroOrange (green), and Mito-tracker (red) (scale bar, 10 μm; *n* = 3 independent experiments). In the merged images, the orange color indicates the co-localization of Mito-tracker (red) and FerroOrange (green). * *p* < 0.05, ** *p* < 0.01, **** *p* < 0.0001. Data were expressed as the mean ± SD. BPD, bronchopulmonary dysplasia; SLC25A28, solute carrier family 25 member 28; CA, citric acid. ACO2, aconitase 2; TOM20, translocase of outer mitochondrial membrane 20; Mito, mitochondrial; Cyto, cytosolic; CON, control; HYP, hyperoxic.

To elucidate the correlation between BPD, OXPHOS, and energy production and conversion pathways, we performed an integrative analysis of the intersection among the corresponding datasets. KEGG enrichment of these intersection datasets s revealed significant enrichment in the pathways related to the regulation of epithelial cell proliferation (Figure 2A,B). AECs dysfunction is a central pathological factor in the development of BPD [24,25]. Thus, we investigated the role of hyperoxic pulmonary injury on AEC. Hyperoxia reduced cell viability (Figure S2A), decreased the mRNA levels of *Spa*, *Spb*, *Spc* and *Spd* (Figure S2B), and reduced Ki-67 positive cells in MLE-12 cells (Figure S2C,D). Moreover, expressions of the key subunits of the ETC (UQCRC1, SDHB, and NDUFS1) were downregulated in hyperoxia-treated MLE-12 cells (Figure S2E,F). Furthermore, mitochondrial metabolomic profiling revealed remarkable citric acid (CA) accumulation in hyperoxia group compared to that in the control (Figure 2C and Figure S2G). Consistent with this result, hyperoxia increased the mitochondrial CA content by almost 2-fold (*p* < 0.01) (Figure 2D), while concurrently reducing the expression (43% decrease, *p* < 0.05) and activity (59% decrease, *p* < 0.01) of aconitase (ACO), an Fe-S-containing protein that functions as the rate-limiting enzyme responsible for the conversion of citrate to isocitrate (Figure 2E,F). As a result, the ATP content in MLE-12 cells was decreased (Figure S2H). These results suggest that hyperoxic stress impairs Fe-S cluster-dependent mitochondrial function. Furthermore, as shown in Figure 2G, hyperoxia downregulated SLC25A28 expression in MLE-12 cells, particularly within mitochondria (Figure 2H). The present study also measured the iron content in different compartments of MLE-12 cells. Decreased mitochondrial and increased cytosolic iron levels were observed in MLE-12 cell under hyperoxic conditions, which was consistent with the in vivo results (Figure 2I–L). Taken together, these findings suggested that the downregulation of SLC25A28 expression and activity contributes to hyperoxia-induced mitochondrial damage in AECs.

### 2.3. SLC25A28 Overexpression Promotes the Proliferation and Migration of MLE-12 Cells

**Figure 3:**
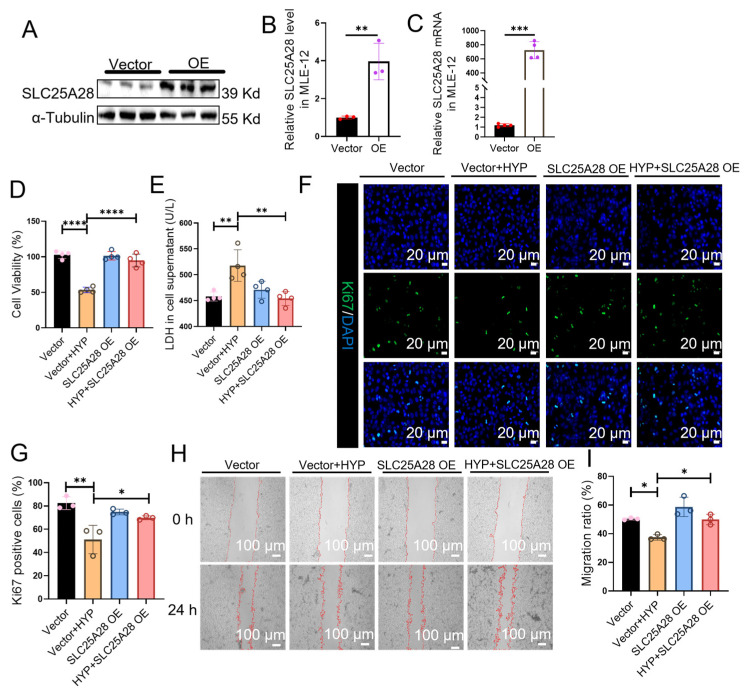
SLC25A28 overexpression promotes the proliferation and migration of MLE-12 cells. (**A**) Representative images of Western blots. (**B**) Relative SLC25A28 protein expression (*n* = 3 independent experiments). (**C**) Relative SLC25A28 mRNA expression (*n* = 4 independent experiments). (**D**) Cell viability by CCK-8 assay (*n* = 4 independent experiments). (**E**) LDH content in MLE-12 cell culture supernatant (*n* = 4 independent experiments). (**F**) Immunofluorescence staining of Ki67 in MLE-12 cells. Ki67 (green) and DAPI (blue) (scale bar, 20 μm). (**G**) Quantitative analysis of Ki67 positive cells (*n* = 3 independent experiments). (**H**) Wound healing assay (scale bar, 100 μm). (**I**) Quantification of the migration ratio (*n* = 3 independent experiments). Data were expressed as the mean ± SD. * *p* < 0.05, ** *p* < 0.01, *** *p* < 0.001, **** *p* < 0.0001. SLC25A28, solute carrier family 25 member 28; LDH, Lactic dehydrogenase; OE, overexpression; HYP, hyperoxic. The red lines outline the edges of the wound. These different color dots are simply part of a standard scatter bar plot. The scatter dots within the bars represent individual biological replicates for each corresponding group.

An SLC25A28 overexpressing plasmid was used to determine the effect of SLC25A28 on the proliferation and migration of MLE-12 cells (Figure 3A–C). SLC25A28 overexpression significantly improved cell viability by 77% (*p* < 0.05), and reduced lactate dehydrogenase (LDH) content by 12% (*p* < 0.01) under hyperoxic conditions compared to the Vector + HYP group (Figure 3D,E). Hyperoxia reduced the number of Ki67-positive cells, whereas SLC25A28 overexpression restored the proportion of Ki67-positive cells by 37% (*p* < 0.05) in hyperoxic conditions (Figure 3F,G). In addition, the wound healing assay revealed that SLC25A28 overexpression enhanced the migration of MLE-12 cells by 33% (*p* < 0.05) under hyperoxic conditions (Figure 3H,I). These results suggest that SLC25A28 overexpression promotes the proliferation and migration of MLE-12 cells under hyperoxic conditions.

### 2.4. Slc25a28 Overexpression Improves Mitochondrial OXPHOS in Hyperoxia-Induced MLE-12 Cells

**Figure 4:**
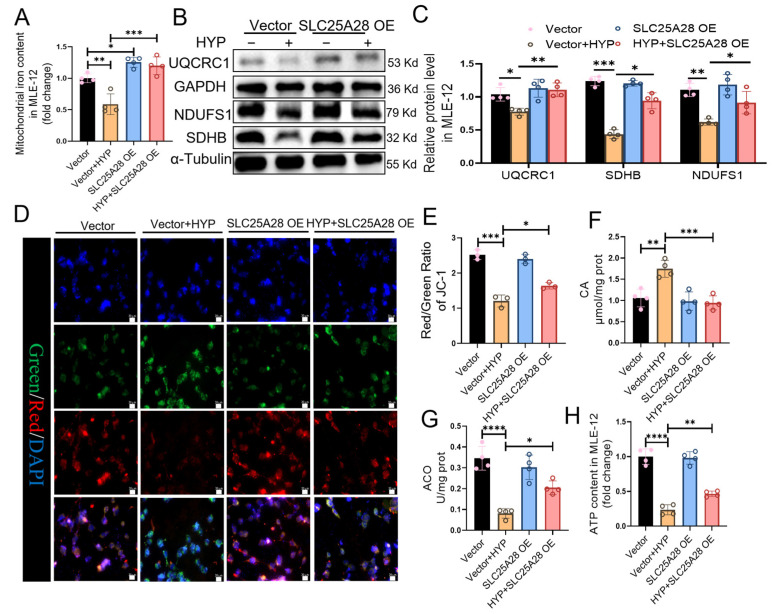
SLC25A28 overexpression improves mitochondrial oxidative phosphorylation system in hyperoxia-induced MLE-12 cells. (**A**) Mitochondrial iron content in MLE-12 cells (*n* = 4 independent experiments). (**B**) Representative images of Western blots. (**C**) Relative protein levels of UQCRC1, NDUFS1 and SDHB (*n* = 4 independent experiments). (**D**) JC-1 staining (scale bar, 20 μm). In the merged images, the pink color indicates the co-localization of JC monomers (green), JC aggregates (red) and DAPI (blue). (**E**) Quantification of mitochondrial membrane potential (*n* = 3 independent experiments). (**F**) Citric acid content in MLE-12 cells (*n* = 4 independent experiments). (**G**) Mitochondrial aconitase activity in MLE-12 cells (*n* = 4 independent experiments). (**H**) ATP content in MLE-12 cells (*n* = 4 independent experiments). * *p* < 0.05, ** *p* < 0.01, *** *p* < 0.001, **** *p* < 0.0001. Data were expressed as the mean ± SD. SLC25A28, solute carrier family 25 member 28; OE, overexpression; HYP, hyperoxic; NDUFS1, NADH: ubiquinone oxidoreductase core subunit S1; SDHB, succinate dehydrogenase complex, subunit B; UQCRC1, ubiquinol-cytochrome c reductase core protein 1; ATP, adenosine triphosphate; CA, citric acid. ACO, aconitase. The different colored bars correspond to the groups indicated in the legend: the black bars represent the Vector group, the orange bars represent the Vector+HYP group, the blue bars represent the SLC25A28 OE group, and the red bars represent the HYP+SLC25A28 OE group.

We further investigated the effect of SLC25A28 on mitochondrial function in MLE-12 cells. As shown in Figure 4A and Figure S3A, SLC25A28 overexpression increased mitochondrial iron content under both normoxic and hyperoxic conditions in MLE-12 cells. Notably, under hyperoxic conditions, SLC25A28 overexpression did not induce cell death compared with Erastin treatment (Figure S3B). Moreover, SLC25A28 overexpression upregulated the expressions of the ETC subunits UQCRC1, SDHB, and NDUFS1 compared to the hyperoxia-treated Vector (Figure 4B,C). SLC25A28 overexpression partially rescued the hyperoxia-induced depolarisation of MMP, as determined by JC-1 staining (Figure 4D,E). Moreover, it reduced CA accumulation and enhanced ACO activity under hyperoxic conditions (Figure 4F,G). Furthermore, ATP production was also increased upon SLC25A28 overexpression under hyperoxic conditions compared to that in the hyperoxia + vector group (Figure 4H). In summary, SLC25A28 overexpression improves mitochondrial OXPHOS under hyperoxic conditions.

### 2.5. Slc25a28 Knockdown Inhibits the Proliferation and Migration of MLE-12 Cells

**Figure 5:**
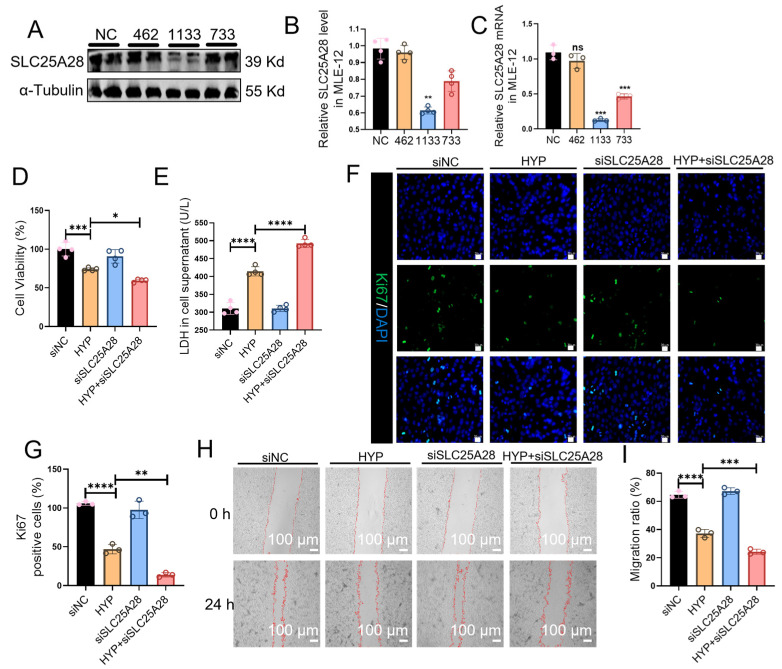
SLC25A28 knockdown inhibits the proliferation and migration of MLE-12 cells. (**A**) Representative images of Western blots. (**B**) Relative SLC25A28 protein expression (*n* = 4 independent experiments). (**C**) Relative SLC25A28 mRNA expression (*n* = 3 independent experiments). (**D**) Cell viability by CCK-8 assay (*n* = 4 independent experiments). (**E**) LDH content in MLE-12 cell culture supernatant (*n* = 4 independent experiments). (**F**) Immunofluorescence staining of Ki67 in MLE-12 cells. Ki67 (green) and DAPI (blue) (scale bar, 20 μm). (**G**) Quantitative analysis of Ki67 positive cells (*n* = 3 independent experiments). (**H**) Wound healing assay (scale bar, 100 μm). (**I**) Quantification of the migration ratio (*n* = 3 independent experiments). The red lines outline the edges of the wound. * *p* < 0.05, ** *p* < 0.01, *** *p* < 0.001, **** *p* < 0.0001, ns, not significant. Data were expressed as the mean ± SD. SLC25A28, solute carrier family 25 member 28; LDH, Lactic dehydrogenase; HYP, hyperoxic; si. short interfering, NC, negative control. The scatter dots within the bars represent individual biological replicates for each corresponding group.

In addition to SLC25A28 overexpression, loss-of-function experiments were performed using SLC25A28-specific siRNA. SLC25A28 siRNA (1133) significantly decreased SLC25A28 mRNA and protein expressions in MLE-12 cells compared with the negative control (NC), confirming the efficiency of SLC25A28 knockdown (Figure 5A–C). As shown in Figure 5D,E, SLC25A28 knockdown further reduced cell viability by 20% (*p* < 0.05) and increased LDH release by 19% (*p* < 0.0001) under hyperoxic conditions. Similarly, SLC25A28 knockdown further reduced the proportion of Ki67 positive cells by 71% (*p* < 0.01) (Figure 5F,G), with a 35% reduction in the migration rate (*p* < 0.001) (Figure 5H,I). These findings suggest that SLC25A28 is essential for the maintenance of AECs proliferation and migration under hyperoxic conditions.

### 2.6. Slc25a28 Knockdown Aggravates Abnormal Mitochondrial OXPHOS in Hyperoxia-Induced MLE-12 Cells

**Figure 6:**
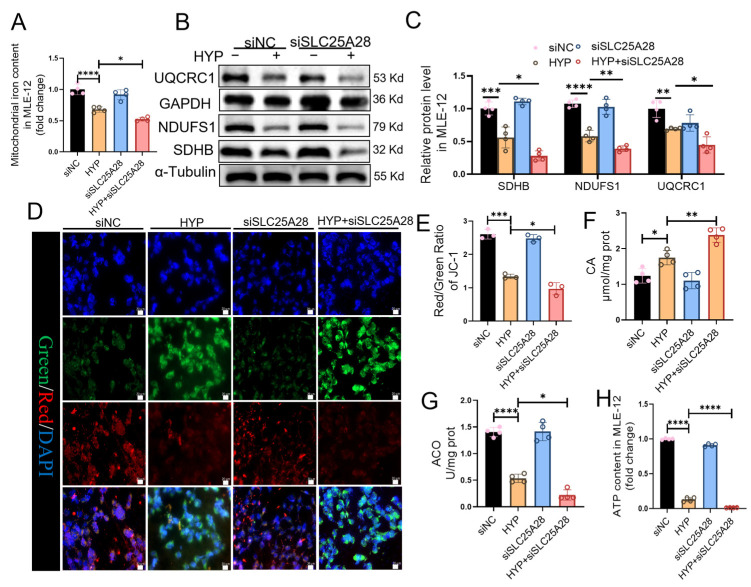
SLC25A28 knockdown aggravates abnormal mitochondrial OXPHOS in hyperoxia-induced MLE-12 cells. (**A**) Mitochondrial iron content in MLE-12 cells (*n* = 4 independent experiments). (**B**) Representative images of Western blots. (**C**) Relative protein levels of UQCRC1, NDUFS1 and SDHB (*n* = 4 independent experiments). (**D**) JC-1 staining (scale bar, 20 μm). (**E**) Quantification of mitochondrial membrane potential (*n* = 3 independent experiments). In the merged images, the pink color indicates the co-localization of JC monomers (green), JC aggregates (red) and DAPI (blue). (**F**) CA content in MLE-12 cells (*n* = 4 independent experiments). (**G**) Mitochondrial aconitase activity in MLE-12 cells (*n* = 4 independent experiments). (**H**) ATP content in MLE-12 cells (*n* = 4 independent experiments). * *p* < 0.05, ** *p* < 0.01, *** *p* < 0.001, **** *p* < 0.0001. Data were expressed as the mean ± SD. SLC25A28, solute carrier family 25 member 28; ATP, adenosine triphosphate; HYP, hyperoxic; si, short interfering; NC, negative control; CA, citric acid; ACO, aconitase; UQCRC1, ubiquinol-cytochrome c reductase core protein 1; NDUFS1, NADH:ubiquinone oxidoreductase core subunit S1; SDHB, succinate dehydrogenase complex, subunit B. The different colored bars correspond to the groups indicated in the legend: the black bars represent the siNC group, the orange bars represent the HYP group, the blue bars represent the siSLC25A28 group, and the red bars represent the HYP+siSLC25A28 group.

As shown in Figure 6A, SLC25A28 knockdown further reduced mitochondrial iron content by 23% (*p* < 0.05) under hyperoxic conditions. However, it did not significantly alter the mitochondrial iron content during normoxia. SLC25A28 knockdown markedly decreased the protein expression of UQCRC1, SDHB, and NDUFS1 in hyperoxia (Figure 6B,C). In addition, SLC25A28 knockdown exacerbated mitochondrial depolarization of MMP under hyperoxic conditions (Figure 6D,E). SLC25A28 knockdown further elevated CA content by 36% (*p* < 0.01), inhibited ACO activity by 58% (*p* < 0.05), and decreased ATP content by 90% (*p* < 0.0001) under hyperoxic conditions (Figure 6F–H).

### 2.7. Iron Supplementation Does Not Increase Slc25a28 Expression Under Hyperoxic Conditions

**Figure 7:**
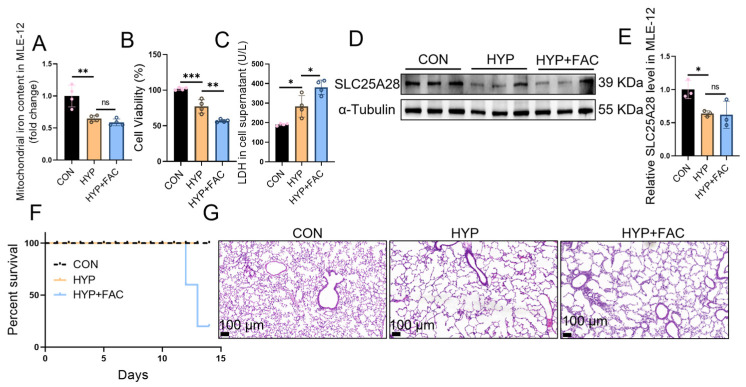
Iron supplementation fails to upregulate the expression of SLC25A28. (**A**) Mitochondrial iron content in MLE-12 cells (*n* = 4 independent experiments). (**B**) Cell viability (*n* = 4 independent experiments). (**C**) LDH content in MLE-12 cell culture supernatant (*n* = 4 independent experiments). (**D**) Representative images of Western blot. (**E**) Relative SLC25A28 protein level in MLE-12 cells (*n* = 3 independent experiments). (**F**) The survival rate of mice (*n* = 6 mice per group). (**G**) Representative hematoxylin and eosin staining of lung tissues (scale bar, 100 μm; *n* = 6 mice per group). * *p* < 0.05, ** *p* < 0.01, *** *p* < 0.001, ns, not significant. Data were expressed as the mean ± SD. SLC25A28, solute carrier family 25 member 28; LDH, lactate dehydrogenase; FAC, ammonium ferric citrate; CON, control; HYP, hyperoxic. The scatter dots within the bars represent individual biological replicates for each corresponding group. The scatter dots within the bars represent individual biological replicates for each corresponding group.

The aforementioned results showed the critical role of SLC25A28 in hyperoxic injury. The SLC25A28 imports iron from the cytosol into mitochondria. Therefore, we further investigated whether supplementation aimed at restoring mitochondrial iron levels would be effective under hyperoxic conditions. Ferric ammonium citrate (FAC), which has been reported to directly restore mitochondrial iron levels was therefore used [26]. However, it failed to increase mitochondrial iron levels under hyperoxic conditions in the present study (Figure 7A). Instead, FAC aggravated cellular damage, as evidenced by a 26% reduction in cell viability (*p* < 0.01) and a 34% increase in LDH content (*p* < 0.05) (Figure 7B,C). We further found that FAC treatment did not significantly alter SLC25A28 expression under hyperoxic conditions (Figure 7D,E). Furthermore, FAC treatment in BPD mice reduced survival rates and failed to mitigate the characteristic alveolar simplification (Figure 7F,G). Collectively, these results further highlight the major role of SLC25A28 in hyperoxic AEC injury.

### 2.8. Slc25a28 Deficiency Further Aggravated Hyperoxia-Induced Lung Injury in BPD Mice

**Figure 8:**
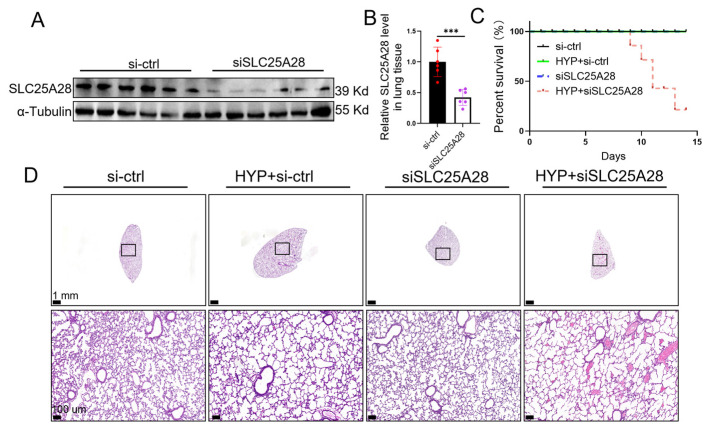
SLC25A28 knockdown aggravated alveolar simplification in BPD mice. (**A**) Representative images of Western blots. (**B**) Relative SLC25A28 protein level (*n* = 6 mice per group). (**C**) The survival rate of mice (*n* = 6 mice per group). (**D**) Representative hematoxylin and eosin staining of lung tissues (scale bar, 100 μm; *n* = 6 mice per group). *** *p* < 0.001, Data were expressed as the mean ± SD. SLC25A28, solute carrier family 25 member 28; HYP, hyperoxic; si, short interfering. The scatter dots within the bars represent individual data points for each corresponding group. The black box indicates the region shown at a higher magnification in the lower panel.

Based on aforementioned findings, we further established an SLC25A28 knockdown mouse model. SLC25A28 siRNA administered intranasally significantly inhibited SLC25A28 expression (>60%) in lung tissues (Figure 8A,B). SLC25A28 knockdown further decreased the survival rate (Figure 8C) and aggravated alveolar simplification in BPD mice (Figure 8D). These results further emphasize the critical role of SLC25A28 in hyperoxia-induced BPD mice.

### 2.9. SS-31 Ameliorates Hyperoxia-Induced Mitochondrial Damage and Alveolar Simplification via Modulating Slc25a28 Expression

**Figure 9:**
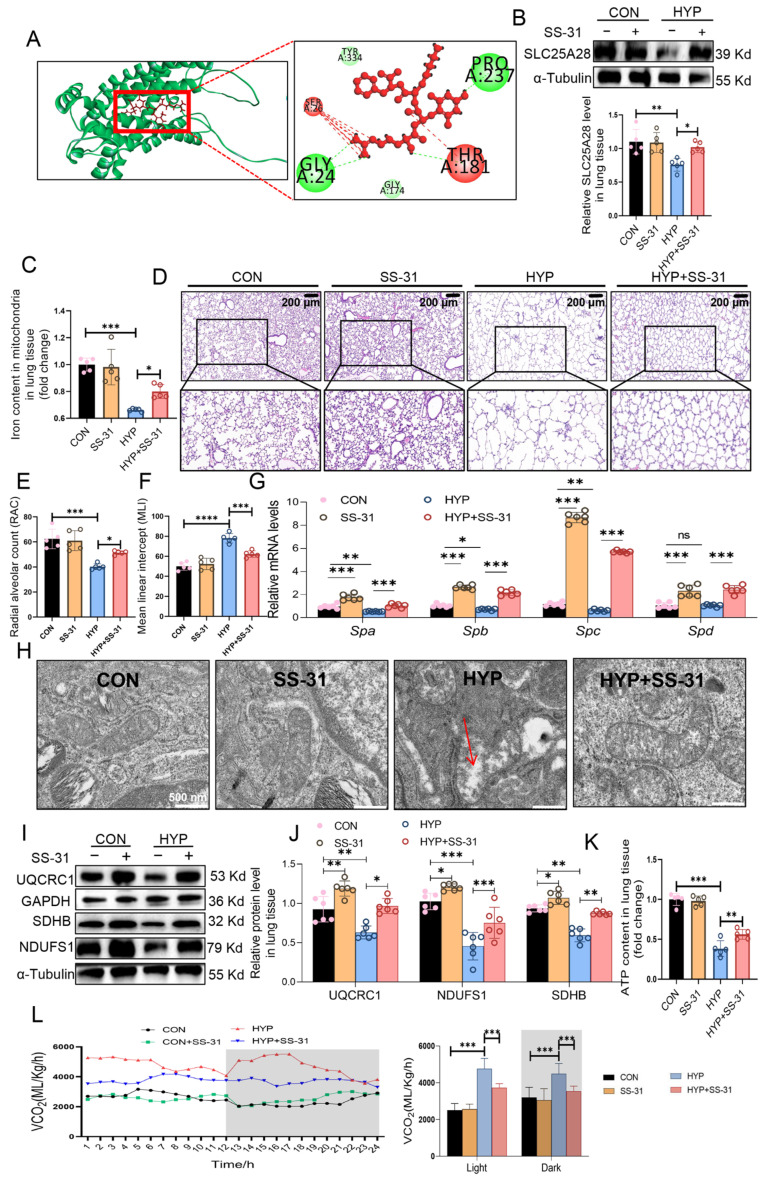
SS-31 mitigates hyperoxia-induced mitochondrial damage and alveolar simplification by upregulating SLC25A28. (**A**) Molecular docking of SS-31 and SLC25A28. (**B**) Relative SLC25A28 protein level (*n* = 5 mice per group). (**C**) Mitochondrial iron content in lung tissues (*n* = 5 mice per group). (**D**) Representative hematoxylin and eosin staining of lung tissues (scale bar, 100 μm). (**E**) RAC evaluation (*n* = 5 mice per group). (**F**) MLI evaluation (*n* = 5 mice per group). (**G**) Relative mRNA levels of *Spa*, *Spb*, *Spc* and *Spd* in lung tissues (*n* = 6 mice per group). (**H**) Transmission electron microscopy images in lung tissue (scale bar, 500 nm; *n* = 6 mice per group). The red arrows indicate the disappearance of mitochondrial cristae. (**I**) Representative images of Western blots. (**J**) Relative protein expressions of NDUFS1, UQCRC1 and SDHB (*n* = 6 mice per group). (**K**) ATP content in lung tissues (*n* = 5 mice per group). (**L**) CO_2_ production (*n* = 4 mice per group). The gray shaded areas indicate the dark phase (nighttime) * *p* < 0.05, ** *p* < 0.01, *** *p* < 0.001, **** *p* < 0.0001, ns, not significant. Data were expressed as the mean ± SD. SLC25A28, solute carrier family 25 member 28; ATP, adenosine triphosphate; RAC, radical alveolar count; MLI, mean linear intercept; UQCRC1, ubiquinol-cytochrome c reductase core protein 1; NDUFS1, NADH:ubiquinone oxidoreductase core subunit S1; SDHB, succinate dehydrogenase complex, subunit; CON, control; HYP, hyperoxic. The scatter dots within the bars represent individual data points for each corresponding group.

Molecular docking analysis revealed that the mitochondrial targeting peptide SS-31 formed hydrogen bonds with specific residues (Gly-A24, Thr-A181, and Pro-A237) in SLC25A28, with a docking score of −10.3 kcal/mol (Figure 9A), indicating strong binding potential. To assess whether SS-31 protects against hyperoxia-induced lung injury by regulating SLC25A28, SS-31 was administered intraperitoneally. SS-31 treatment significantly preserved SLC25A28 protein abundance and activity in lung tissues (Figure 9B,C), suggesting that SS-31 may partially maintain SLC25A28 abundance through this putative interaction. Furthermore, it markedly attenuated alveolar simplification in BPD mice, with and a significant increase in RAC by 28% (*p* < 0.05) and a decrease in MLI by 20% (*p* < 0.01) (Figure 9D–F). In addition, SS-31 increased the mRNA expression of *Spa*, *Spb*, *Spc*, and *Spd* under both normoxic and hyperoxic conditions (Figure 9G). As shown in Figure 9H, SS-31 administration improved the mitochondrial ultrastructure in the lung tissues of BPD mice, as evidenced by intact mitochondrial membranes, the absence of swelling, and compact cristae. Consistent with these morphological improvements, SS-31 treatment markedly upregulated the expression of key ETC subunits, including SDHA, SDHB, and NDUFS1 (Figure 9I,J). Furthermore, SS-31 significantly increased ATP content in the lung tissues of BPD mice (Figure 9K), indicating enhanced OXPHOS. In addition, SS-31 treatment reversed hyperoxia-induced elevation in CO_2_ production (Figure 9L). These results suggest that SS-31 modulates SLC25A28 and protects against hyperoxia-induced alveolar simplification.

### 2.10. SS-31 Promotes Proliferation via Activating SLC25A28 in Hyperoxia-Induced MLE-12 Cells

**Figure 10:**
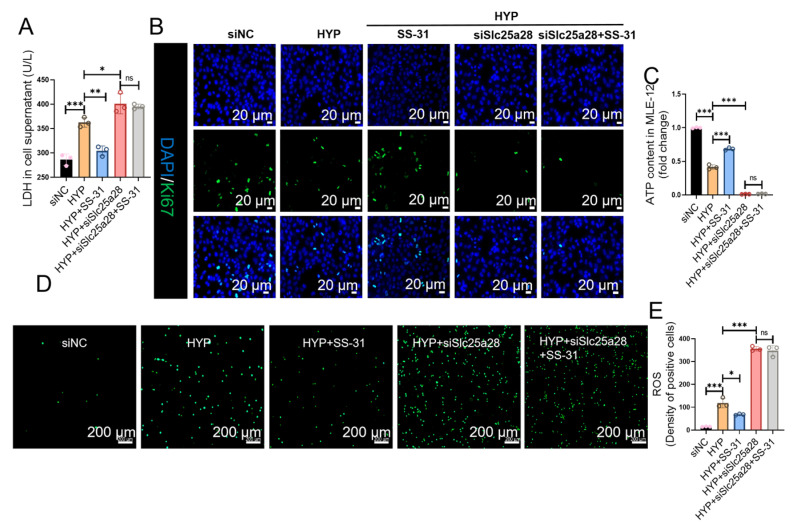
SS-31 promotes proliferation via activating SLC25A28 in hyperoxia-induced MLE-12 cells. (**A**) LDH content in MLE-12 cell culture supernatant (*n* = 3 independent experiments). (**B**) Immunofluorescence staining of Ki67. Ki67 (green) and DAPI (blue). (scale bar, 20 μm; *n* = 3 independent experiments). (**C**) ATP content in MLE-12 cells (*n* = 3 independent experiments). (**D**) Intracellular ROS was detected using the DCFH-DA fluorescent probes (scale bar, 200 μm). (**E**) Quantification of ROS content (*n* = 3 independent experiments). * *p* < 0.05, ** *p* < 0.01, *** *p* < 0.001, ns, not significant. Data were expressed as the mean ± SD. SLC25A28, solute carrier family 25 member 28; LDH, lactate dehydrogenase; ATP, adenosine triphosphate; ROS, reactive oxygen species; HYP, hyperoxic; si, short interfering; NC, negative control. The scatter dots within the bars represent individual biological replicates for each corresponding group.

SLC25A28 siRNA was used to determine whether the beneficial effects of SS-31 against hyperoxia-induced AECs depend on SLC25A28. The HYP + SS-31 + siSLC25A28 group failed to reduce LDH content and increase the number of Ki67 positive cells compared to the HYP + siSLC25A28 group (Figure 10A,B). In addition, SLC25A28 knockdown reduced ATP content and increased reactive oxygen species (ROS) production in hyperoxia-stimulated MLE-12 cells. The antioxidant and mitochondrial protective effects of SS-31 were offset after SLC25A28 siRNA treatment (Figure 10C–E). These results indicate that the beneficial effect of SS-31 against hyperoxia-induced AECs injury are dependent on the modulation of SCL25A28.

## 3. Discussion

As the most common complication in premature infants, BPD is a global health issue for which no effective therapies are currently available. Therefore, it is essential to identify new therapeutic targets and develop novel treatment approaches to improve alveolar development. In this study, we identified SLC25A28 as a novel target for the treatment of hyperoxia-induced BPD. The key findings are summarized as follows: (1) Under hyperoxic conditions, SLC25A28 expression and activity were downregulated and mitochondrial OXPHOS was impaired both in vivo and in vitro. (2) SLC25A28 overexpression restored mitochondrial OXPHOS, and promoted the proliferation and migration of hyperoxia-treated MLE-12 cells. In contrast, SLC25A28 knockdown exerted opposite effects. (3) SLC25A28 knockdown aggravated alveolar simplification in BPD mice. (4) The mitochondrial-targeted peptide SS-31 attenuated alveolar simplification in BPD mice by modulating SLC25A28. This study elucidates the protective role of SLC25A28 in hyperoxia-induced alveolar simplification in neonatal mice and provides insights into the mechanisms by which mitochondrial function is restored.

The function of AECs is to produce and recycle surfactant to maintain alveolar surface tension [27,28]. Pulmonary surfactants play an indispensable role during the final phases of lung maturation. By reducing surface tension at the air-liquid interface, these agents facilitate alveolar patency and prevent atelectasis, thereby ensuring long-term structural stability [29]. Alveolar regeneration is primarily directed by AECs and disruption of AECs homeostasis contributes to the pathogenesis of BPD [30,31]. In this study, we found AEC injury in lung tissues of BPD mice and in hyperoxia-stimulated MLE-12 cells, which was consistent with previous studies [32]. It has been reported that AECs have abundant mitochondria as an energy source for the production and secretion of pulmonary surfactant [33]. Mitochondria, the primary site for energy production, also serve as the major source of ROS [34]. Impaired mitochondrial OXPHOS has been observed in hyperoxia-stimulated AECs, as characterized by the reduced ATP content, disrupted MMP, and impaired mitochondrial ETC [35,36]. Ultimately, such impaired mitochondrial energetics further contributes to the onset and progression of BPD [37]. Moreover, mitochondrial function can predict early mortality and poor pulmonary outcomes in preterm infants with BPD [38]. In our prior study, persistent mitochondrial damage was detectable even in the offspring of hyperoxia-exposed maternal mice [39]. Here, we also found abnormal mitochondrial OXPHOS and damaged mitochondrial ultrastructure under hyperoxic conditions. Accumulating studies have demonstrated that targeting mitochondria constitutes a viable strategy to mitigate hyperoxia-induced BPD [40,41]. Collectively, targeting mitochondrial bioenergetics in alveolar epithelial cells could be a fundamental mechanism and a promising strategy for preventing alveolar developmental failure in premature infants.

Iron is an essential trace element in mammalian cells. Current research on iron and BPD has mainly focused on ferroptosis [42,43]. Ferroptosis is characterized by iron accumulation and excessive lipid peroxidation. Experiments in a BPD mouse model showed the accumulation of iron in the lung [44]. It is not surprising that ferroptosis is recognized as a driving factor in BPD because oxidative damage is a hallmark of hyperoxia-induced pulmonary damage [45]. It has been proposed that excessive iron supplementation in infants with very low birth weight increases the risk of developing BPD [46]. However, other studies have reported no increased risk of BPD with iron supplementation [47]. Therefore, further research is required to elucidate the specific regulatory mechanisms underlying ferroptosis in BPD. In this study, we observed altered iron distribution under hyperoxic conditions, with cytosolic iron overload and mitochondrial iron deficiency. This specific compartmentalization suggests that ferroptosis might not be the sole contributor to cell death. Although cytosolic iron promotes oxidative damage, the implications of mitochondrial iron depletion warrant particular attention. Iron is an indispensable cofactor for the ETC and heme synthesis [6,7]. The observed mitochondrial iron deficiency likely leads to ETC dysfunction, resulting in a state of mitochondrial bioenergetic failure. Therefore, the pulmonary injury in BPD could be a synergistic outcome of ferroptotic lipid peroxidation (driven by cytosolic iron) and metabolic collapse (driven by mitochondrial iron deprivation). Moreover, we found that supplementation of FAC aggravated hyperoxic pulmonary injury. We attribute this to the possibility that FAC exacerbates the elevated cytosolic iron levels, thereby fueling ferroptosis, without effectively restoring mitochondrial iron homeostasis or reversing the bioenergetic crisis. These results highlight the important role of mitochondrial iron deficiency in hyperoxia-induced mitochondrial energy failure in BPD.

Mitochondrial iron mainly participates in the synthesis of Fe-S proteins [48], which are essential for cellular vitality and are involved in key biochemical processes such as energy metabolism, DNA synthesis and repair [49]. Recent studies have shown that hyperoxia impairs mitochondrial Fe-S cluster proteins, thereby disrupting mitochondrial ETC function [12]. NDUFS1 and SDHB are both Fe-S cluster-containing subunits that are essential for mitochondrial OXPHOS. In the present study, we observed that hyperoxia reduced the abundance of NDUFS1 and SDHB, accompanied by decreased aconitase activity, a functional indicator of mitochondrial Fe-S cluster integrity. The reduced levels of NDUFS1 and SDHB are likely associated with impaired Fe-S cluster biogenesis and/or stability. However, because the mRNA expression and protein turnover of NDUFS1 and SDHB were not directly examined in the current study, we could not determine whether their reduction was primarily caused by decreased synthesis or increased degradation. This question warrants further investigation in future studies. SLC25A28 is a mitochondrial high-affinity iron importer. Abnormal function of SLC25A28 can lead to mitochondrial iron metabolism disorders and mitochondrial dysfunction, which are involved in the occurrence of diseases such as brain injury and cancer [50,51]. In this study, both the mRNA and protein expression levels of SLC25A28 decreased in lung tissue from BPD mice. Notably, SLC25A28 overexpression restored aconitase activity, whereas SLC25A28 knockdown further impaired it, supporting the idea that SLC25A28 is required for maintaining Fe-S cluster-dependent mitochondrial homeostasis under hyperoxic stress. As expected, SLC25A28 knockdown further aggravated mitochondrial OXPHOS and exacerbated AECs injury, SLC25A28 overexpression promoted mitochondrial OXPHOS and attenuated hyperoxia-induced AECs damage, and SLC25A28 knockdown aggravated alveolar simplification in BPD mice. These results highlight the crucial role of SLC25A28 in the maintenance of mitochondrial OXPHOS throughout BPD progression.

SS-31 is a synthetic tetrapeptide designed to selectively target mitochondria and restore mitochondrial bioenergetics. It can effectively reduce levels of O_2_^−^, H_2_O_2_, hydroxyl radical and peroxynitrite both in vitro and in vivo. Additionally, it can increase ATP levels to maintain MMP [52,53]. Consistent with these findings, we also found that SS-31 increased ATP levels and improved energy metabolism in hyperoxia-exposed lung tissues. Furthermore, it preserved physiological ROS levels. As we know, maintaining moderate ROS levels is essential for physiological signaling. The anti-oxidative effect of SS-31 was thought to inhibit the excessive ROS production stemming from the ETC. This is a significant benefit of SS-31 compared to traditional antioxidants, as the excessive use of traditional antioxidants may deplete physiological ROS levels [54]. Interestingly, the expressions of UQCRC1, NDUFS1 and SDHB in lung tissues were increased even under normal conditions in this study. UQCRC1, NDUFS1 and SDHB are subunits of Complex I, Complex II and Complex III respectively, all located within the ETC. It has been reported that SS-31 can enhance the efficiency of the ETC [55,56]. Thus, the upregulated genes involved in mitochondrial complexes may provide a material basis for the enhancement of ETC function.

This study also has several limitations. First, our in vivo experiments relied on a single, continuous hyperoxia exposure protocol (85% O_2_) in neonatal mice. While this is a widely accepted model for BPD, human BPD is a complex, multi-factorial disease driven by fluctuating oxygen levels, mechanical ventilation, and infections. The conclusions of this study are based solely on a neonatal mouse model, and whether they are applicable to other species remains to be determined. Although SLC25A28 decreased under hyperoxic conditions both in vivo and in vitro, the upstream mechanisms regulating this reduction remain unclear. Second, SS-31 preserved SLC25A28 protein abundance and attenuated alveolar simplification in BPD mice. The interaction between SS-31 and SLC25A28 was primarily predicted using molecular docking analysis. Direct physical binding assays, such as co-immunoprecipitation, pull-down or surface plasmon resonance are required in future studies to definitively validate this interaction. In addition, the precise mechanism through which SS-31 modulates SLC25A28 expression, whether by preventing its degradation or enhancing its synthesis remains to be elucidated. There also remains a significant translational gap regarding the clinical application of SS-31. The optimal dosing strategies, pharmacokinetics, and safe delivery routes (e.g., systemic administration versus inhalation) for extremely premature infants have yet to be determined, warranting rigorous clinical and pharmacokinetic investigations in the future. Furthermore, only four mice per group (*n* = 4) were used to determine metabolic parameters using a metabolic cage. Using the G power 3.1.9.7 software, we set the α error probability at 0.05, and calculated statistical power based on the sample size. Although the power in each group reached the expected level (power > 0.8), greater attention should be paid to improving the rigor and reliability of the experimental design in future studies.

## 4. Materials and Methods

### 4.1. Reagents

SS-31 (MW: 639.79, HPLC = 98.83%) was obtained from Selleck Chemicals (#S9803; Houston, TX, USA), FAC (#HY-B1645) and erastin (#HY-15763) were purchased from MCE (Monmouth Junction, NJ, USA). FerroOrange (#F347) was purchased from Dojindo (Kumamoto, Japan). Mito-Tracker Red (#M22425) was purchased from Invitrogen (Durham, NC, USA). The kits for LDH (#A020-2) and hematoxylin and eosin (H&E) staining (#D006-1) were obtained from the Nanjing Jiancheng Bioengineering Institute (Nanjing, China). Ferrous determination kit (#ADS-W-QT027) was purchased from Jiangsu Meimian Industrial Co., Ltd. (Yancheng, Jiangsu, China). The CA Content Assay Kit (#BC2155) and ACO Activity Assay Kit (#BC4485) were obtained from Solarbio Biotechnology (Beijing, China).

### 4.2. Animals

In total, 18 pregnant C57BL/6 mice (7–9 weeks old, weighing 20–25g) were obtained from SPFbiotech Co., Ltd. (Nanjing, China). All animal experiments were approved by the Ethics Committee of Jiangnan University (JN.No20240915c0180301[466] and date of approval 15 September 2024). The mice were maintained in SPF conditions at 22–25 °C and a relative humidity of 40–70%, following a 12 h light/dark cycle. All the animals had free access to food and water.

FiO_2_ = 0.85 was used to establish the hyperoxia-induced BPD model as previously reported [57,58]. Briefly, newborn mice (within 12 h of birth, including both male and female pups) were pooled and randomized to dams and exposed to normoxia (FiO_2_ = 0.21) or hyperoxia (FiO_2_ = 0.85) for 14 days. The hyperoxic pups were fed by dams in a sealed plexiglass chamber, and the oxygen concentration was continuously monitored with a monitor (XBS-03S, AIPUINS, Hangzhou, China). The inside of the chamber was kept at atmospheric pressure, and dams were rotated every 24 h between normoxic and hyperoxic conditions to avoid oxygen poisoning.

To determine the effect of SLC25A28 on BPD mice, SLC25A28 siRNA was used to establish a loss-of-function model. SLC25A28 siRNA was synthesized and conjugated with a cholesterol residue at the 3′-end and full-chain methoxy modification (GenePharma, Shanghai, China). The anti-SLC25A28 siRNA sequences are listed in Table 1. Newborn mice were randomly divided into four groups (*n* = 6 per group): mice maintained under normoxia and administered negative control siRNA (si-ctrl), mice maintained under normoxia and administered SLC25A28 siRNA (siSLC25A28), mice exposed to hyperoxia and administered negative control siRNA (HYP + si-ctrl), and mice exposed to hyperoxia and administered SLC25A28 siRNA (HYP + siSLC25A28). SLC25A28 siRNA (5 nM) or the negative control was administered intranasally every other day from postnatal days (PN) 1 to PN 14 [59].

To evaluate the protective effect of FAC against BPD, neonatal mice were randomly divided into three groups (*n* = 6 per group): normoxia (CON), hyperoxia (HYP) and hyperoxia + FAC (HYP + FAC). FAC (15 mg/kg) [60] was intraperitoneally injected daily for seven consecutive days starting from postnatal days 7–13 (PN7-13).

To examine the protective effect of SS-31 against BPD, all newborn mice were randomly divided into four groups (*n* = 6 per group): mice maintained under normoxia (CON), mice maintained under normoxia and administered SS-31 (SS-31), mice exposed to hyperoxia (HYP), and mice exposed to hyperoxia and administered SS-31 (HYP + SS-31). SS-31 (5 mg/kg) [22] was intraperitoneally injected into the mice daily for three consecutive days from PN11 to 14.

For the animal experiments, our sample size (*n* = 6 per group) was determined based on standard practices in similar studies within the field [44,61,62] and strictly adheres to the 3Rs principle (Replacement, Reduction, and Refinement) for the ethical use of animals. The goal was to minimize the number of animals used while ensuring sufficient statistical power to detect biologically meaningful differences. Therefore, we chosen *n* = 6 mice per group for the in vivo experiments to ensure robustness.

Importantly, the humane endpoints established for this study included: (1) Weight loss exceeding 20% of the initial body weight; (2) Inability to eat or drink freely; (3) Severe lethargy or a sustained hunched posture; (4) Signs of severe pain or distress that could not be relieved. We confirm that during this study, no mice met the pre-defined humane endpoint criteria that would have necessitated early intervention and euthanasia prior to the scheduled experimental endpoint. All animals were euthanized at the pre-determined experimental endpoint. On PN14. the mice were eventually anesthetized by inhalation of 2% isoflurane and sacrificed via cervical dislocation. Death was confirmed by the absence of a heartbeat, cessation of breathing, and loss of the corneal reflex. The euthanasia of fetal mice in our study was performed according to the Guidelines for the Euthanasia of Animals from American Veterinary Medical Association (AVMA) (2020 Edition).

### 4.3. Survival Rate of Mice

The general health of the mice (*n* = 6 per group) was monitored daily from PN0 to PN14. The survival status was recorded using a binary system, where 0 represented survival and 1 represented death. Deaths occurring during this experiment were recorded as part of the survival analysis. We confirmed that no animals required euthanasia based on the predefined humane endpoint criteria prior to the scheduled end of the experiment.

### 4.4. Comprehensive Laboratory Animal Monitoring System

After acclimation for 24 h, metabolic parameters were continuously measured for 24 h using a comprehensive laboratory animal monitoring system (CLAMS) (Columbus Instruments, OH, USA) as we previously reported [63].

### 4.5. Histopathological Evaluation of Lung Tissues

The left lungs were collected and fixed in 4% paraformaldehyde for 48 h, then embedded in paraffin, and cut into 4 μm thick slices. Sections were subjected to H&E staining (Nanjing Jiancheng, Nanjing, China). The RAC and MLI were calculated as described previously [64].

### 4.6. TEM Evaluation

Lung samples were fixed in a specialized electron microscope fixative (Servicebio, Wuhan, China), exposed to osmium, dehydrated in ethanol, and dehydrated with polymerized epoxy. After embedding, thin sections were cut by using ultramicrotome (Leica, Stuttgart, Germany) and mounted on 200-mesh copper grids. The samples were then stained with lead citrate and uranyl acetate. TEM images of mitochondrial cristae and morphology were obtained using a Tecnai G220 TWIN transmission electron microscope.

### 4.7. Cell Culture and Treatment

The MLE-12 cells were maintained in DMEM (GIBCO, Carlsbad, CA, USA) supplemented with 10% fetal bovine serum (FBS) (GIBCO, Grand Island, NY, USA) and 1% penicillin–streptomycin solution (GIBCO, Grand Island, NY, USA) in a Thermo Scientific incubator (Waltham, MA, USA) with 5% CO_2_ at 37 °C. Considering that isolated cells lack the systemic defense and are directly exposed to hyperoxia, a moderately high oxygen tension (70% O_2_) was selected for the in vitro hyperoxia model, as previously reported [65,66,67]. Briefly, MLE-12 cells were pre-incubated with SS-31 (20 μM) or FAC (50 μM) [26] for 30 min, then they were exposed to hyperoxia (70% O_2_) stimulation for 24 h. The oxygen concentration was continuously monitored with electrochemical sensor (PUHE Scientific, Wuxi, China).

In our in vitro experiments, at least 3 independent biological replicates was used. This is a common and established sample size in cell biology literature for preliminary mechanistic studies [44,68]. Each experiment included internal technical replicates to ensure assay precision. The consistency of the observed effects across all three independent replicates strongly supports the reproducibility of our findings.

### 4.8. Slc25a28 Overexpression Plasmid Transfection

An empty plasmid (Vector) or a SLC25A28 overexpression plasmid (SLC25A28 OE) was obtained from GenePharma (Shanghai, China). MLE-12 cells were seeded in 6-well plates at a density of 1 × 10^5^ cells/mL. After 50% confluent, the MLE-12 cells were transfected with the SLC25A28 overexpression plasmid or Vector (1.5 μg) according to the manufacturer’s instructions (#C10511-05; RiboBio, Guangzhou, China). Briefly, each plasmid was diluted in serum-free medium to prepare a DNA mixture. A separate mixture containing the transfection reagent was prepared in the same medium. After incubation at room temperature for 10 min, the two mixtures were combined and incubated further to allow complex formation. The resulting transfection complexes were then added dropwise to the cultured MLE-12 cells. The medium was changed after 24 h, and then the cells were subsequently exposed to hyperoxia (70% O_2_) or normoxia (21% O_2_) for another 24 h.

### 4.9. Slc25a28 si-RNA Transfection

SLC25A28 was knocked down with SLC25A28 siRNA in MLE-12 cells. After 50% confluent, the MLE-12 cells were transfected with the negative control (NC) (20 μM) or SLC25A28 siRNA (20 μM) for 24 h. The cells were then exposed to hyperoxia (70% O_2_) or normoxia (21% O_2_) for 24 h to examine the effect of SLC25A28 under hyperoxic conditions. To determine whether SS-31 targets SCL25A28, the transfected cells were then subjected to SS-31 treatments and hyperoxia exposure for 24 h. The siRNA sequences including NC and SLC25A28 siRNAs, are presented in Table 2.

### 4.10. FerroOrange and Mito-Tracker Staining

FerroOrange (F347; Tokyo, Japan) was used to detect intracellular Fe^2+^ levels and Mito-Tracker Red (M22425; Invitrogen, Carlsbad, CA, USA) was used to mark the mitochondrial. The cells were seeded in 12-well plates at a density of 1 × 10^5^ cells/well. They were incubated with FerroGreen solution (1 μM/L) and Mito-Tracker Red solution (1:1000) for 30 min. Next, the cells were observed using a fluorescence confocal microscopy (Carl Zeiss LSM880).

### 4.11. Cell Viability Assay

A CCK-8 assay (C6005; NCM Biotech, Shanghai, China) was used to detect the viability of MLE-12 cells. MLE-12 cells (5 × 10^4^/mL) were seeded in 96-well plates. A CCK-8 solution (10 μL) was added to treated cells, followed by incubation at 37 °C for 1 h. Optical density was measured at an absorption wavelength of 450 nm.

### 4.12. Wound Healing Assay

MLE-12 cells were seeded into 6-well plates. After SLC25A28 overexpression or siRNA transfection, a straight line was drawn with a 100 μL sterile micropipette tip in the center of each well and photographed (0 h). The cells were cultured under normal or hyperoxic conditions. After 24 h, MLE-12 cells were observed under an inverted microscope (Axio Vert A1, Carl Zeiss, Oberkohen, Germany).

### 4.13. The MMP Evaluation

The MMP of MLE-12 cells was measured using the fluorescent dye JC-1 (#C2003S; Beyotime, Nanjing, China). Briefly, after stimulation, MLE-12 cells were treated with JC-1 for 30 min at 37 °C, and JC monomers (488 nm) and JC aggregates (570 nm) were visualized using a fluorescence microscope (Olympus, Tokyo, Japan).

### 4.14. The ROS Content Measurement

Intracellular ROS production in MLE-12 cells was measured using the fluorescent probe 2′,7′-dichlorodihydrofluorescein diacetate (DCFH-DA; #CA1410; Solarbio). Briefly, the cells were loaded with 10 μM DCFH-DA and incubated at 37 °C for 30 min in the dark. Subsequently, fluorescence was visualized and captured using a Zeiss Axio Imager 2 fluorescent microscope (Carl Zeiss AG).

### 4.15. Mitochondrial Non-Targeted Metabolomics

Mitochondria were isolated from MLE-12 cells using a mitochondrial extraction kit (#C3601; Beyotime). Next, the mitochondria were immediately resuspended in a methanol: water (4:1, *v*/*v*) to halt the metabolic reaction. Metabolomic data were analysed by OE Biotech Company (Shanghai, China). The raw data were processed using Progenesis QI for peak alignment, normalization, and metabolite identification. Multivariate statistical analyses, including PCA and OPLS-DA, were performed to discriminate group differences and identify significant metabolites. The OPLS-DA model was validated using 7-fold cross-validation and 200 permutation tests to prevent overfitting. Differential metabolites were selected based on a VIP score > 1 from the OPLS-DA model and a *p*-value < 0.05 using a two-tailed Student’s *t*-test.

### 4.16. Immunofluorescence Staining

Lung sections slides or MLE-12 cells were prepared and permeabilized with 1% Triton X-100 and blocked with 5% BSA for 2 h. The slides were probed with primary antibodies against ki67 (1:300, ab279653; abcam, Cambridge, UK), SPC (1:300, 10774-1-AP; proteintech, Wuhan, China) and SLC25A28 (1:200, 680417; Zenbio, Chengdu, China) followed by Alexa Fluor 488-conjugated secondary antibody (Cat#33106ES60; Yeasen, Shanghai, China) or Alexa Fluor 594-conjugated secondary antibody (Cat#33112ES60; Yeasen, Shanghai, China). The slides or the cells were then incubated in DAPI (#P0131; Beyotime, Naning, China) for 5 min and the images were captured using a Zeiss Axio Imager Z2 fluorescence microscope (Carl Zeiss, Oberkochen, Germany).

### 4.17. Mitochondria Isolation and Iron Assay

Mitochondrial components were isolated from MLE-12 cells and lung tissues using mitochondrial isolation kits (#C3601 and #3606; Beyotime,). Briefly, samples were homogenized in a PMSF-containing isolation solution on ice and centrifuged at 1000× *g* (4 °C, 10 min). The resulting supernatant was further centrifuged at 12,000× *g* (4 °C, 10 min) to collect the mitochondrial pellet. Mitochondrial iron content was determined by measuring the absorbance at 562 nm using a Bio-Tek Epoch microplate reader (BioTek; Agilent Technologies, Inc., Santa Clara, CA, USA).

### 4.18. Biochemical Assays

The LDH levels in the serum and cell culture supernatants were measured using commercial kits (Nanjing Jiancheng Bioengineering Institute, Nanjing, China). The intracellular CA content and ACO activity were determined according to the manufacturer’s instruction (Solarbio, Beijing, China). The Absorbance was measured at 450 nm, 545 nm, and 240 nm respectively using a microplate reader (Bio-Tek Epoch, Winooski, VT, USA).

### 4.19. Real-Time Fluorescent Quantitative PCR (RT-qPCR)

Total RNA from lung tissues or MLE-12 cells was extracted using TRIzol reagent (Vazyme, Nanjing, China) and then reverse-transcribed to cDNA using PrimeScript RT Reagent Kit (Yeasen, Shanghai, China). Targeted mRNA quantification was measured by RT-qPCR using SYBR Premix Ex TaqTM (Yeasen Biotechnology; 11201ES08) with a LightCycler^®^ 480 detection PCR system (Roche, Foster City, CA, USA). The PCR amplification protocol consisted of an initial denaturation step at 95 °C for 5 min, followed by 40 cycles of denaturation at 95 °C for 10 s, annealing at 55 °C for 20 s, and extension at 72 °C for 20 s. Relative quantification was performed using the 2^−ΔΔCt^ method, with β-actin serving as the reference gene. The primer sequences are listed in Table 3. 

### 4.20. Western Blotting

Total protein was extracted from lung tissues and MLE-12 cells using RIPA lysate containing protease inhibitors. The protein extracts were separated by 12% SDS-PAGE and transferred to polyvinylidene fluoride (PVDF) membrane (Millipore Corp, Bedford, MA, USA). The membranes were blocked with 5% skim milk at room temperature for 2 h and then incubated with the indicated primary antibodies overnight at 4 °C, and subsequently incubated with horseradish peroxidase (HRP)-conjugated secondary antibodies. The following primary antibodies were used: anti-SLC25A28 (1:1000, 680417; Zenbio), anti-TOM20 (1:5000, 11802-1-AP; Proteintech), anti-UQCRC1 (1:500, 8235014; Zenbio), anti-ACO2 (1:2000, 11134-1-AP; Proteintech), anti-NDUFS1 (1:500, R389132; Zenbio), anti-SDHB (1:500, R381845; Zenbio), anti-GAPDH (1:5000, 60004-1-Ig; Proteintech) and anti-α-Tubulin (1:2000, 11224-1-AP; Proteintech).

### 4.21. Bioinformatics Analysis

Three gene sets related to BPD, OXPHOS, and energy production and conversion were retrieved from the GeneCards database. The intersections were identified using a Venn diagram. Overlapping genes were subsequently subjected to KEGG pathway enrichment analysis using the STRING database.

### 4.22. Molecular Docking

The structure of SS-31 (CID:11764719) was downloaded from the PubChem drug database, and the structure of SLC25A28 (ID:PRO_0000235255) was obtained from UniPort. Pretreatment was performed using a Discovery Studio Visualiser (v18.1.0.18287; San Diego, CA, USA) for water removal and hydrogenation. The docking conformations of SS-31 and SLC25A28 were simulated and visualized using Discovery Studio Visualizer. Results with higher scores and better conformations were selected as molecular docking results.

### 4.23. Statistical Analysis

All cellular and molecular experiments were independently repeated for at least 3 times. Statistical data are expressed as means ± standard deviation (SD). The Shapiro–Wilk test was utilized to assess data normality. For normally distributed quantitative data, comparisons between two independent groups were performed using an unpaired, two-tailed Student’s *t*-test. For comparisons involving three or more groups, the homogeneity of variance was first evaluated using the Brown–Forsythe test. If the variances were equal, a one-way analysis of variance (ANOVA) followed by Tukey’s post hoc test was applied. If the variances were unequal, Welch’s ANOVA followed by Dunnett’s T3 post hoc test was employed for multiple comparisons. Analyses were performed using GraphPad Prism (version 8.0.2; GraphPad Inc., San Diego, CA, USA). *p* < 0.05 was considered statistically significant.

## 5. Conclusions

Our findings reveal the protective role of SLC25A28 in hyperoxia-induced alveolar simplification in neonatal mice, a mechanism that enhances mitochondrial OXPHOS via improving mitochondrial iron utilization. These findings indicate that SLC25A28 is a potential target for BPD, and that SS-31 is a candidate drug for BPD.

## Figures and Tables

**Table 1:** anti-SLC25A28 siRNA sequences.

Gene	Sense (5′→3′)	Antisense (5′→3′)
*siNC*	/i2OMeU//i2OMeU//i2OMeC//i2OMeU//i2	/i2OMeA//i2OMeC//i2OMeG//i2OMeU//i2
	OMeC//i2OMeC//i2OMeG//i2OMeA//i2O	OMeG//i2OMeA//i2OMeC//i2OMeA//i2O
	MeA//i2OMeC//i2OMeG//i2OMeU//i2OM	MeC//i2OMeG//i2OMeU//i2OMeU//i2OM
	eG//i2OMeU//i2OMeC//i2OMeA//i2OMe	eC//i2OMeG//i2OMeG//i2OMeA//i2OMe
	C//i2OMeG//i2OMeU/TT	G//i2OMeA//i2OMeA/TT
*siSLC25A28*	/i2OMeG//i2OMeC//i2OMeC//i2OMeA//i2	/i2OMeA//i2OMeC//i2OMeA//i2OMeC//i2
	OMeU//i2OMeC//i2OMeG//i2OMeC//i2O	OMeA//i2OMeG//i2OMeA//i2OMeC//i2O
	MeA//i2OMeU//i2OMeG//i2OMeG//i2OM	MeC//i2OMeA//i2OMeU//i2OMeG//i2OM
	eU//i2OMeC//i2OMeU//i2OMeG//i2OMe	eC//i2OMeG//i2OMeA//i2OMeU//i2OMe
	U//i2OMeG//i2OMeU/TT	G//i2OMeG//i2OMeC/TT

**Table 2:** siRNA sequence.

siRNA Name	Strand	Sequence (5′–3′)
Negative Control	Sense	UUCUCCGAACGUGUCACGUdTdT
	Anti-sense	ACGUGACACGUUCGGAGAAdTdT
SLC25A28-462	Sense	GCUCUCUGGAGAAUCAUGAdTdT
	Anti-sense	UCAUGAUUCUCCAGAGAGCdTdT
SLC25A28-1133	Sense	GCCAUCGCAUGGUCUGUGUdTdT
	Anti-sense	ACACAGACCAUGCGAUGGCdTdT
SLC25A28-733	Sense	CCGCGUGACAGACUGUGUUdTdT
	Anti-sense	AACACAGUCUGUCACGCGGdTdT

**Table 3:** Primer sequences for quantitative real-time PCR.

Gene	Forward Primer (5′-3′)	Reverse Primer (5′-3′)
*β-actin*	GGCTGTATTCCCCTCCATCG	CCAGTTGGTAACAATGCCATGT
*Ndufs1*	AGGATATGTTCGCACAACTGG	TCATGGTAACAGAATCGAGGGA
*Sdha*	GGAACACTCCAAAAACAGACCT	CCACCACTGGGTATTGAGTAGAA
*Sdhb*	AATTTGCCATTTACCGATGGGA	AGCATCCAACACCATAGGTCC
*Uqcrc1*	AGACCCAGGTCAGCATCTTG	GCCGATTCTTTGTTCCCTTGA
*Uqcrc2*	AAAGTTGCCCCGAAGGTTAAA	GAGCATAGTTTTCCAGAGAAGCA
*Atp5a*	TCTCCATGCCTCTAACACTCG	CCAGGTCAACAGACGTGTCAG
*Atp5b*	GGTTCATCCTGCCAGAGACTA	AATCCCTCATCGAACTGGACG
*Dph1*	GGCAGAGGTCGCATCTCTC	ATCCACAATAGTGCAGGCAAA
*Dph2*	AAGACCTGGACCGCGTGTA	ATCTTAGCTCCTGTGACTTCCT
*Ercc2*	ACCCGGAGCAGTTCTCCTAC	GGTCACCTCCAGCGGATAAG
*Ppat*	GCGAGGAATGTGGTGTGTTTG	TTTAGGCACTGCACTCCCATC
*Spa*	GAGGAGCTTCAGACTGCACTC	AGACTTTATCCCCCACTGACAG
*Spb*	CTGCTTCCTACCCTCTGCTG	CTTGGCACAGGTCATTAGCTC
*Spc*	ATGGACATGAGTAGCAAAGAGGT	CACGATGAGAAGGCGTTTGAG
*Spd*	AAGGTCCACGGGGTGAGAA	TTTGCCTTGAGGTCCTATGTTC
*Slc25a28*	AGCATTGCGTGATGTACCCG	CCTGTTGCTGTGACGTTCA
*Fxn*	TTGAAGACCTTGCAGACAAG	AGCCAGATTTGCTTGTTTGG
*Iscu*	ATGAAAAGGGGAAGATTGTGG	AAGCAGCTGCTGTGACTG
*Nfs1*	CTGCGCGTTGTAGATCATGG	AGTTGACAAGGTATGGGAGCAT
*Slc25a37*	CCTACTCCACGATGCAGTAATG	AGTGAATTGACTGGAAGGGGATA

## Data Availability

Dataset available on request from the authors. The metabolomic data generated from the present study have been uploaded and are publicly available at the China National Center for Bioinformation under the accession code OMIX015107 (https://share.cncb.ac.cn/hfB3dL96FP/OMIX015107/) and accessed on 1 March 2026.

## Supplementary Materials

The following supporting information can be downloaded at: https://www.mdpi.com/article/10.3390/ijms27083357/s1.

## Abbreviations

ATP, adenosine triphosphate; α-KGDH, α-ketoglutarate dehydrogenase; BPD, bronchopulmonary dysplasia; ETC, electron transport chain; GSH, Glutathione; IDH, isocitrate dehydrogenase; LDH, Lactate Dehydrogenase; MDA, Malondialdehyde; MDH, malate dehydrogenase; MMP, mitochondrial membrane potential; OXPHOS, oxidative phosphorylation system; ROS, reactive oxygen species; SOD, Superoxide dismutase; TEM, transmission electron microscopy.
