# Supplementary material for: SLC25A28 Ameliorates Hyperoxic Lung Injury by Improving Mitochondrial Oxidative Phosphorylation in Alveolar Epithelial Cells
Source: Int J Mol Sci. 2026 Apr 8;27(8):3357. doi: 10.3390/ijms27083357 (PMC13116717; doi:10.3390/ijms27083357)
Supplement: Supplementary file 1 [file ijms-27-03357-s001.zip › ijms-4219083-supplementary.pdf]

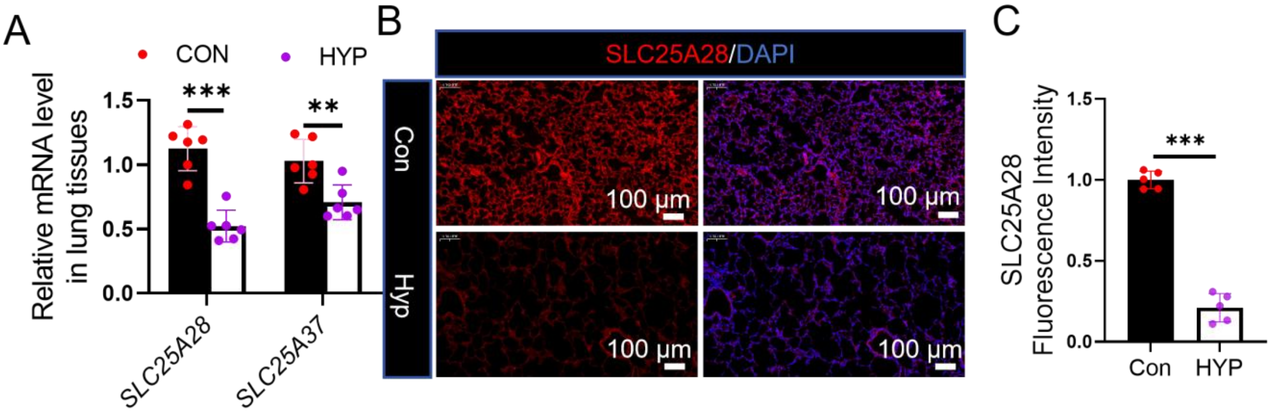

Figure S1. SLC25A28 downregulation is associated with impaired mitochondrial function in lung tissues of BPD mice. (A) Relative mRNA levels of SLC25A28 and SLC25A37 in lung tissues (n=6 mice per group). (B) Immunofluorescence staining of SLC25A28 in lung tissues. SLC25A28 (red) and DAPI (blue; scale bar, 100 μm). (B) Quantitative analysis of SLC25A28 immunofluorescence staining (n=5 mice per group). **P<0.01, ***P<0.001. Data were expressed as the mean ± SD. SLC25A28, solute carrier family 25 member 28; SLC25A37, solute carrier family 25 member 37; CON, control; HYP, hyperoxic.

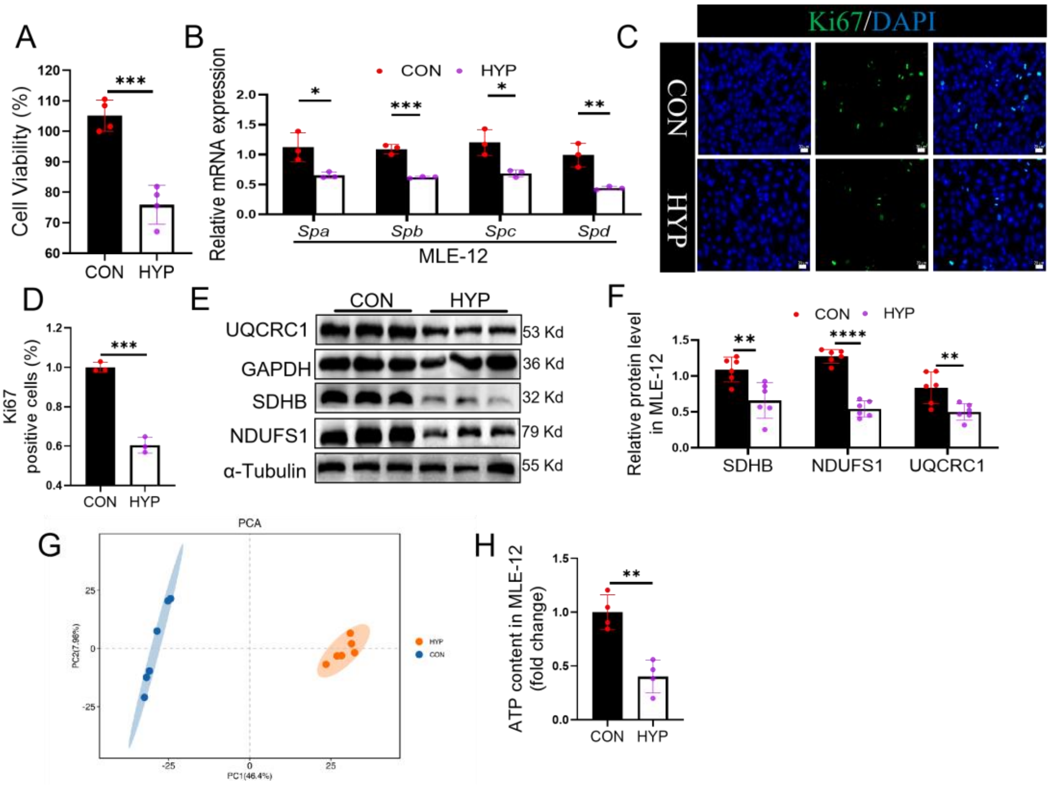

Figure S2. Hyperoxia resulted in SLC25A28 downregulation and mitochondrial damage in MLE-12 cells. (A) Cell viability (n=4 independent experiments). (B) Relative mRNA expression of Spa, Spb, Spc and Spd expression in MLE-12 cells (n=3). (C) Immunofluorescence staining of Ki67 in MLE-12 cells. Ki67 (green) and DAPI (blue; scale bar, 20 μm). (D) Quantification of Ki67 positive cells (n=3 independent experiments). (E) Representative images of western blots. (F) Relative protein levels of NDUFS1, SDHB and UQCRC1 (n=6 independent experiments). (G) Mitochondrial principal component analysis of two groups (n=6 per group). (H) ATP content in MLE-12 cells (n=4 independent experiments). *P <0.05, **P<0.01, ***P<0.001, ****P<0.0001. Data were expressed as the

mean ± SD. UQCRC1, ubiquinol-cytochrome c reductase core protein 1; NDUFS1, NADH:ubiquinone oxidoreductase core subunit S1; SDHB, succinate dehydrogenase complex, subunit B; ATP, adenosine triphosphate; CON, control; HYP, hyperoxic.

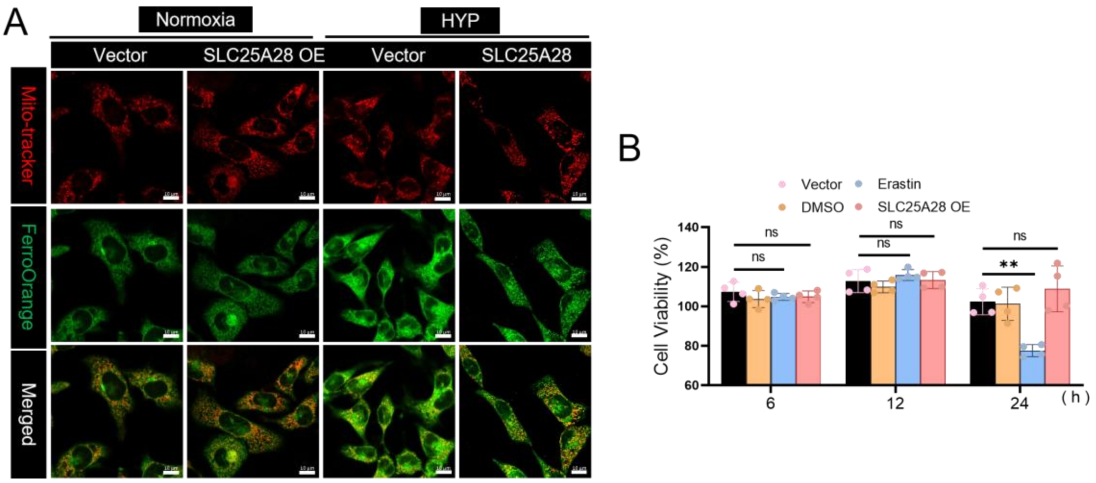

Figure S3. SLC25A28 overexpression enhanced mitochondrial iron content in MLE-12 cells. (A) FerroOrange and Mito-tracker staining showed that the mitochondrial iron contents. FerroOrange (green) and Mito-tracker (red; scale bar, 10 μm; n=3 independent experiments). (B) CCK-8 assay (n=4 independent experiments). **P<0.01, ns, not significant. Data were expressed as the mean ± SD. SLC25A28, solute carrier family 25 member 28; HYP, hyperoxic
